# 

**Published:** 1899-09

**Authors:** 


### BOOKS RECEIVED.

The Treatment of Pelvic Inflammations Through the Vagina. By William H. Pryor, M. D., Professor of Gynecology, New York Polyclinic; Consulting Surgeon, City (Charity) Hospital, etc. 12mo, pp. 248. With 110 illustrations. Philadelphia: W. B. Saunders. 1899.

Mt. Sinai Hospital Reports. Volume I. For 1898. Edited for the Medical Board by Paul F. Mundé, M. D. New York: Stettiner Bros. 1899.

Annual Report of the Supervising Surgeon-General of the Marine Hospital Service of the United States for the fiscal year 1898, Centennial Year. 8vo, pp. 855. Washington: Government Printing Office. 1899.

The Hygiene of Transmissible Diseases: Their Causation, Modes of Dissemination and Methods of Prevention. By A. C. Abbott, M. D., Professor of Hygiene and Director of the Laboratory of Hygiene, University of Pennsylvania. Octavo volume of 311 pages. Illustrated. Price, $2.00 net. Philadelphia: W. B. Saunders, Publisher.

General Pathology, or the Science of the Causes, Nature and Course of the Pathological Disturbances which occur in the living subject. By Dr. Ernst Ziegler, Professor of Pathological Anatomy and of General Pathology at the University of Freiburg in Breisgau. Translated from the ninth revised German edition, by Drs. Theo. Dunham, E. M. Foote, Philip H. Hiss, Jr., Walter B. James, Wm. G. Le Boutillier and Matthias Nicoll, Jr., of New York; Dr. B. Meade Bolton, of Philadelphia, Pa., and Drs. Leonard Woolsey Bacon, Jr., John S. Ely and R. A. McDonnell, of New Haven, Conn. Editor, Dr. Albert H. Buck, of New York. Complete in one octavo volume of 621 pages, profusely illustrated by 544 wood engravings in black and numerous colors, and lithographic plate. Bound in extra muslin at $5.00 net, and in brown sheep at $5.75 net. New York: William Wood & Co. 1899.

A Text-Book of Pharmacology and Therapeutics, or the Action of Drugs in Health and Disease. For the use of Students and Practitioners of Medicine. By Arthur R. Cushny, M. A., M. D., Aberd. Professor of Materia Medica and Therapeutics in the University of Michigan Medical Department, Ann Arbor. In one octavo volume of 728 pages, with 47 engravings. Cloth, $3.75 net. Philadelphia and New York: Lea Brothers & Co. 1899.

A Text-Book of Diseases of the Nose and Throat. By D. Braden Kyle, M. D., Clinical Professor of Laryngology and Rhinology, Jefferson Medical College, etc., etc. Octavo, pp. 646. With 175 illustrations, 23 of them in colors. Price, $4.00, cloth; sheep and one-half morocco, $5.00, net. Philadelphia: W. B. Saunders. 1899.

International Clinics. A Quarterly of Clinical Lectures on Medicine, Neurology, Surgery, Gynecology and Obstetrics, Ophthalmology, Laryngology, Pharyngology, Rhinology, Otology and Dermatology, and Specially Prepared Articles on Treatment and Drugs. By professors and lecturers in the leading medical colleges of the United States, Germany, Austria, France, Great Britain and Canada. Edited by Judson Daland, M. D. (Univ. of Penna.), Philadelphia, Instructor in Clinical Medicine and Lecturer on Physical Diagnosis in the University of Pennsylvania; Assistant Physician to the Hospital of the University of Pennsylvania, etc.; Fellow of the College of Physicians of Philadelphia. Volume II. Ninth series. 1899. Octavo, pp. x.—310. Philadelphia: J. B. Lippincott Co. 1899.

Over 1,000 Prescriptions or Favorite Formulæ of Various Teachers, Authors and Practicing Physicians. The whole being carefully indexed, and including most of the newer remedies. Cloth, 300 pages; postpaid, $1.00. Detroit, Mich.: The Illustrated Medical Journal Co., Publishers. 1899.

Transactions of the Medical Association of Central New York. Thirty-first annual meeting held at Auburn, N. Y., October 18, 1898. William C. Krauss, M. D., Editor. Volume V. Buffalo: Buffalo Medical Journal Print. 1899.

American Pocket Medical Dictionary. Edited by W. A. Newman Dorland, A. M., M. D., Assistant Obstetrician to the Hospital of the University of Pennsylvania, etc. Containing the pronunciation and definition of over 26,000 of the terms used in medicine and the kindred sciences, along with over 60 extensive tables. Second edition, revised. Price, $1.25 net. Philadelphia: W. B. Saunders. 1899.

Handy Book of Medical Progress; A Lexicon of the Recent Advances in Medical Science. By Charles Warrenne Allen, M. D., and Jacob Sobel, M. D. One volume, post, 8vo. Extra buckram, $1.50 net. William Wood & Company. 1899.

Surgical Nursing. A Compilation of the Lectures upon Abdominal Surgery, Gynecology and General Surgical Conditions and Procedures, delivered to the classes in the training school for nurses connected with the Woman's Hospital of Philadelphia. By Anna M. Fullerton, M. D., Clinical Professor of Gynecology in the Woman's Medical College; Obstetrician, Gynecologist and Surgeon to the Woman's Hospital. Third edition, revised and enlarged, with 69 illustrations. Price, $1.00. Philadelphia: P. Blakiston's Son & Co. 1899.

